# What affects the selection of diverting ileostomy in rectal cancer surgery: a single-center retrospective study

**DOI:** 10.1186/s12893-024-02316-3

**Published:** 2024-01-23

**Authors:** Zhen Wang, Yuchen Guo, Shuang Li, Liang He, Yinquan Zhao, Quan Wang

**Affiliations:** Department of Gastrocolorectal Surgery, General Surgery Center, The First Hospital of Jilin University, Changchun, 130021 China

**Keywords:** Pelvimetry, Rectal cancer, Diverting ileostomy, Laparoscopic surgery

## Abstract

**Background:**

The selection of diverting ileostomy (DI) is controversial. This study aimed to explore the factors affecting the selection of diverting ileostomy (DI) following laparoscopic low anterior resection for rectal cancer.

**Methods:**

This retrospective, case-control study included patients who underwent laparoscopic-assisted sphincter-saving surgery for mid-low rectal cancer from January 2019 to June 2021. Univariate and multivariate analyses were performed on the patient’s clinicopathological characteristics and pelvic dimensions measured by abdominopelvic electron beam computed tomography.

**Results:**

A total of 382 patients were included in the analysis, of which 182 patients (47.6%) did not undergo DI, and 200 patients (52.4%) underwent DI. The univariate analysis suggested that male sex (*p* = 0.003), preoperative radiotherapy (*p* < 0.001), patients with an anastomosis below the levator ani plane (*p* < 0.001), the intertuberous distance (*p* < 0.001), the sacrococcygeal distance (*p* = 0.025), the mid pelvis anteroposterior diameter (*p* = 0.009), and the interspinous distance (*p* < 0.001) were associated with performing DI. Multivariate analysis confirmed that preoperative radiotherapy (*p* = 0.037, odds ratio [OR] = 2.98, 95% confidence interval [CI] = 1.07–8.30), anastomosis below the levator ani plane (*p* < 0.001, OR = 7.09, 95% CI = 4.13–12.18), and the interspinous distance (*p* = 0.047, OR = 0.97, 95% CI = 0.93-1.00) were independently associated with performing DI.

**Conclusion:**

Pelvic parameters also influence the choice of DI. According to this single-center experience, patients with a shorter interspinous distance, particularly narrow pelvic with an interspinous distance of < 94.8 mm, preoperative radiotherapy, and anastomosis below the levator ani plane, prefer to have a DI and should be adequately prepared by the physician.

## Introduction

Anastomotic leakage (AL) is a serious complication of laparoscopic surgery for rectal cancer, with its incidence reportedly ranging from 2 to 19%, with AL-related mortality ranging from 0.8 to 27% [1, 2]. AL affects the quality of life through diverting stoma creation incompetence or affects the patient’s oncological outcome [3, 4]. Diverting ileostomy (DI) was widely employed in low rectal surgery to decrease the risk of this complication. Although several ongoing studies suggest that DI might not decrease the incidence of AL, it still lowers the severity of abdominopelvic infection due to AL and decreases the rate of secondary surgery [5, 6]. However, several studies have reported adverse outcomes concerning diverting stoma creation [7]; therefore, mastering appropriate ileostomy indications can maximise patient benefits. Nevertheless, no clear international consensus on the indication of DI in low rectal cancer exists [8], and the optimal time for selecting a DI is controversial [9–11]. This study identified and analysed the factors influencing a clinician’s choice of DI and clarified the benefits of DI in anus-preserving surgery for mid-low rectal cancer.

## Methods

This retrospective, case-control study included patients who underwent laparoscopic-assisted surgery at the Department of Gastrocolorectal Surgery,General Surgery Center, The First Hospital of Jilin University, China, from January 2019 to June 2021. We obtained preoperative baseline data, intraoperative information, postoperative complications, and follow-up information from these patients. The Ethics Committee of The First Hospital of Jilin University approved this study.

Due to surgeon factor would affect surgical process [12], the same experienced surgical team at our center performed all procedures in order to eliminate bias caused by different experience. All surgeons have the same level of experiece. Mechanical bowel preparation was routinely performed preoperatively. All surgeries were performed laparoscopically, and all cases underwent mobilisation of the splenic flexure and high ligation of the inferior mesenteric vessels. End-to-end anastomosis was performed, low anterior resection (LAR) or transanal total mesorectum excision (taTME) was selected according to the patient’s tumour location, and pelvic drainage was performed, followed by selective prophylactic ileostomy,all diverting ileostomy were double-barreled ileostomy.

The inclusion criteria were as follows: (1) a diagnosis of rectal adenocarcinoma established by pathological examination; (2) tumour margin ≤ 10 cm from the anal verge; (3) patients who underwent laparoscopic LAR or ultra-low anterior resection; (4) the stoma type was terminal ileum stoma double-barreled ileostomy. The exclusion criteria were as follows: (1) the presence of multiple intestinal tumours; (2) distant metastasis observed during preoperative surgery (liver, lung, etc.); (3) those who underwent emergency surgery due to intestinal obstruction, bleeding, or perforation; (4) combined organ resection (bladder, prostate, etc.).

All patients underwent laboratory tests and abdominal computed tomography (CT) to assess their preoperative conditions. The clinicopathological variables included in the study were as follows: sex, age at operation, height, weight, body mass index, history of diabetes, history of abdominal surgery, postoperative hospital stay, tumour height from the anal verge, distance from the anal verge of the anastomosis, preoperative albumin/globulin (> 1.2 vs. <1.2), preoperative haemoglobin (< 90 g/L vs. ≥90 g/L), history of preoperative radiotherapy, American Society of Anaesthesiologists physical status classification grade, DI, and blood loss (< 100 mL vs. ≥100 mL),opreation time,number of stapled firings,air test. AL within 30 days after surgery and its grade was recorded. The levator ani plane was defined as the anatomical plane of the origin of the levator ani muscle. The relationship between the anastomosis and the levator ani plane was assessed intraoperatively, the tumour size was measured postoperatively, and the longest diameter of the cross-section was recorded. Air test was confirmed by the transanal air instillation, and any leaks were repaired by intraoperative suturing. The pathological stage (I vs. II vs. III) was recorded based on the pathology reports, and preoperative radiotherapy was recommended for patients with low rectal cancer (T3/T4N^+^) according to the National Comprehensive Cancer Network guidelines,long course radiotherapy which combined with chemotherapy in radiotherapy interval,and 50 Gy were administered.

**Fig. 1:**
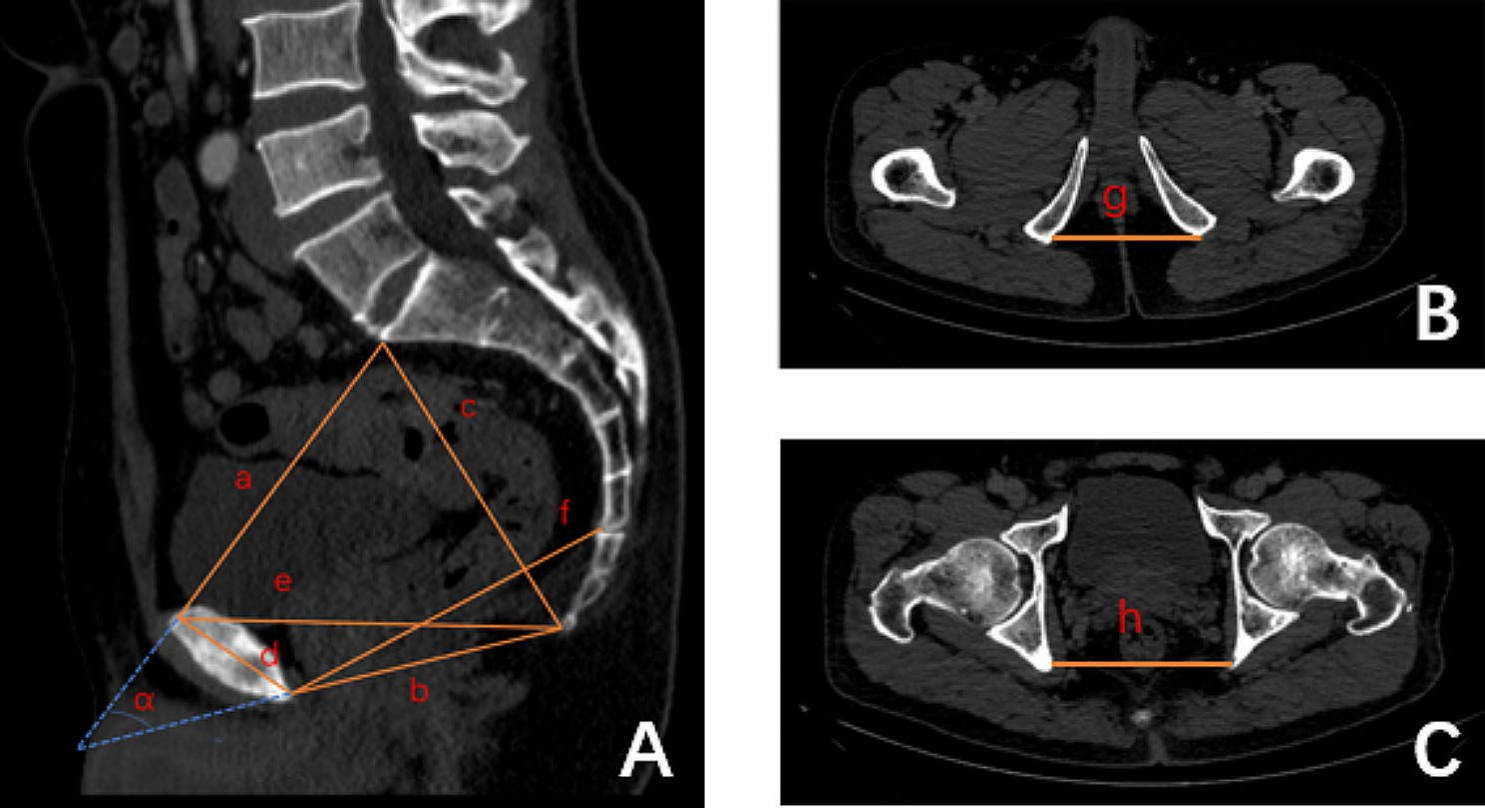
Pelvimetry measured by computed tomography. **a:** Midsagittal position, anteroposterior diameter of the pelvic inlet; **b:** anteroposterior diameter of the pelvic outlet; **c:** sacrococcygeal distance; **d:** pubic symphysis height; **e:** distance between the superior margin of the pubic symphysis and the coccyx; **f:** midpelvic anteroposterior diameter; α angle between the pelvic inlet and extension of the anteroposterior diameter of the pelvic outlet B, C transverse position, **g:** intertuberous distance; **h:** interspinous distance

Pelvimetry was measured using a three-dimensional reconstruction of the contrast-enhanced CT scans having a slice thickness of 5 mm and an interslice distance of 5 mm. Two imaging specialists measured the pelvic data, and the two specialists were blinded to the patient’s clinical information. Mid-sagittal and axial sections of the pelvis were used to obtain the pelvic dimensions. The following nine factors were measured in this study. The intertuberous distance was defined as the distance between the two sides of the ischial tuberosity. The sacrococcygeal distance was defined as the distance from the sacral promontory to the sacrococcygeal junction. The midpelvic anteroposterior diameter was defined as the distance from the middle of the inferior border of the pubic symphysis to the median border of the sacrococcygeal region. The height of the pubic symphysis was defined as the distance from the middle of the superior border of the pubic symphysis to the middle of the inferior border of the pubic symphysis. The interspinous distance was defined as the distance between the ischial spines on both sides. The anteroposterior diameter of the pelvic inlet was defined as the length from the middle of the upper edge of the pubic symphysis to the center of the anterior edge of the sacrococcygeal promontory. The anteroposterior diameter of the pelvic outlet was defined as the distance from the middle of the lower edge of the pubic symphysis to the caudal apex. The distance from the upper edge of the pubic symphysis to the coccyx was defined as the distance from the upper midline of the pubic symphysis to the caudate apex. The angle α was defined as the angle between the anteroposterior diameter of the pelvic inlet and the extension of the anteroposterior diameter of the pelvic outlet [13–15]. Pelvic dimensions were measured as shown in Fig. 1 using CT images for pelvic measurements. AL was assessed within 30 days after surgery based on the clinical and imaging findings. AL was assessed based on the definition of the International Rectal Cancer Group as an interruption and defect in the integrity of the intestinal wall at the colon-rectal or colon-anal anastomosis, resulting in the communication of the intraluminal and extraluminal compartments and pelvic abscess beside the anastomotic site. AL is divided into three grades, which are as follows: grade A did not require a change in the treatment modality, grade B required intervention but did not require surgery, and grade C required surgical treatment [16]. Herein, AL requiring clinical intervention (grade B leakage and grade C leakage) were diagnosed based on a range of clinical symptoms, such as fever, abdominal or pelvic pain, vaginal or drainage tube discharge of gas or faeces, pelvic abscess, or peritonitis and required specific treatment, including local drainage or abdominal surgery [17]. When AL is suspected, abdominal CT should be performed to confirm the diagnosis. At the same time, the 30-day postoperative mortality and postoperative hospital stay were also statistically analysed.

### Statistical analysis

Statistical analysis was performed using SPSS 26.0 statistical software. Data are expressed as the number of cases and percentage (%), and the chi-square test and Fisher’s exact test were used for comparing data. Normally distributed measurement data are expressed as the mean ± standard deviation, and the Student’s t-test was used for comparison between groups. Non-normally distributed measurement data are expressed as the median and quartiles (P25, P75), and between-group differences were evaluated using the U test. Univariate analysis was used for relevant variables, and variables with *p* < 0.05 were included in the multivariate logistic regression before collinearity testing of variables. Variables with a variance inflation factor of > 5 were excluded from the equation, and cut-off values were determined using the area under the receiver operating characteristic (ROC) curve for continuous variables with *p* < 0.05 in the regression equation. Statistical significance was set at *p* < 0.05.

## Results

We included 382 patients in this study, of which 182 (47.6%) underwent DI and 200 (52.4%) did not undergo DIPatients with low rectal cancer (≤ 5 cm) were 132 (34.6%) and with middle rectal cancer (≤ 10 > 5) were 250 (65.4%). The clinicopathological characteristics and surgical information of patients in each group are presented in Table 1. The pelvic parameter measurement data are presented in Table 2. The univariate analysis of these variables revealed that male sex (*p* = 0.003), preoperative radiotherapy (*p* < 0.001), anastomosis below the levator ani plane (*p* < 0.001), the intertuberous distance (*p* < 0.001), the sacrococcygeal distance (*p* = 0.025), the midpelvic anteroposterior diameter (*p* = 0.009), and the interspinous distance (*p* < 0.001) were associated with DI.

**Table 1:** Univariate and multivariate analyses of the characteristics of the 382 patients

Variable	Level	Without DI(*n* = 182)	With DI(*n* = 200)	Univariate analysis pvalue	multivariate analysis OR(95%CI) pvalue
Sex	Male	110	149	*0.003	1.30(0.64–2.68) 0.472
	Female	72	51		
Age(yr)	<65	100	131	*0.035	0.92(0.57–1.49) 0.733
	≥ 65	82	69		
BMI(kg/m²)	<28	164	167	0.058	
	≥ 28	18	33		
Diabetes	No	152	161	0.444	
	Yes	30	39		
History of abdominal surgery	No	170	181	0.971	
	Yes	12	13		
ALB/GLB	≥ 1.2	153	163	0.508	
	<1.2	29	37		
Preoperative hemoglobin(g/L)	≥ 90	181	197	0.362	
	<90	1	3		
Preoperative radiotherapy	No	176	170	*<0.001	2.98(1.07–8.30) 0.037
	Yes	6	30		
ASA	1	4	1	0.344	
	2	150	167		
	3	28	32		
Blood loss(ml)	<100	168	190	0.279	
	≥ 100	14	10		
Anastomosis located below the levator ani plane	Yes	23	113	*<0.001	7.09(4.13–12.18)<0.001
	No	159	87		
Maximum tumor diameter(cm)	<5	133	153	0.441	
	≥ 5	49			
Pathological stage	I	40	62	0.062	
	II	62	50		
Surgical procedure	III LAR TaTME	80 161 21	88 102 98	<0.001	
Operation time(min)	>180	60	83	0.085	
	≤ 180	122	117		
Tumor height(cm)	≤ 10>5	158	92	<0.001	
	≤5	24	108		
Number of stapled firings	Handsewn	20	37	0.119	
	≤ 2>0	101	103		
	>2	61	60		
Air Test	Positive	10	18	0.189	
	Negative	172	182		

***OR*** odds ratio,***CI*** confidence-intervial,***BMI*** body mass index,***ALB/GLB*** albumin/globulin,***ASA*** American Society of Anaesthesiologists. *P* values are derived from two-tailed tests.*Values are statistical significance (*p*<0.05)

**Table 2:** Univariate and multivariate analyses of the pelvic measurements in 382 patients

Variable(mm)	Without DI (*n* = 182)	With DI (*n* = 200)	Univariate analysis pvalue	multivariate analysis OR(95%CI) pvalue
intertuberous distance	106.00(97.98,116.13)	99.00(94.03,109.93)	*<0.001	0.99(0.97–1.03) 0.971
sacrococcygeal distance	119.01 ± 12.23	121.93 ± 13.06	*0.025	1.02(0.99–1.03) 0.130
midpelvic anteroposterior diameter	109.00(103.88,115.00)	107.05(100.00,112.40)	*0.009	0.98(0.95–1.01) 0.206
pubic symphysis height	38.40 ± 3.90	38.11 ± 4,28	0.498	
interspinous distance	97.40(89.98,107.33)	91.15(86.00,99.58)	*<0.001	0.97(0.93-1.00) 0.047
anteroposterior diameter of the pelvic inlet	108.87 ± 9.77	108.09 ± 12.48	0.499	
anteroposterior diameter of the pelvic outlet	89.71 ± 9.72	88.90 ± 8.35	0.385	
Angle α	43.00(39.15,48.03)	43.10(38.90,47.18)	0.954	
superior margin of pubic symphysis to coccyx distance	117.66 ± 9.60	116.13 ± 8.97	0.107	

***OR*** odds ratio,***CI*** confidence-intervial.,***Angle α*** between the pelvic inlet and extension of the anteroposterior diameter of the pelvic outlet. *P* values are derived from two-tailed tests.*Values are statistical significance (*p*<0.05)

**Fig. 2:**
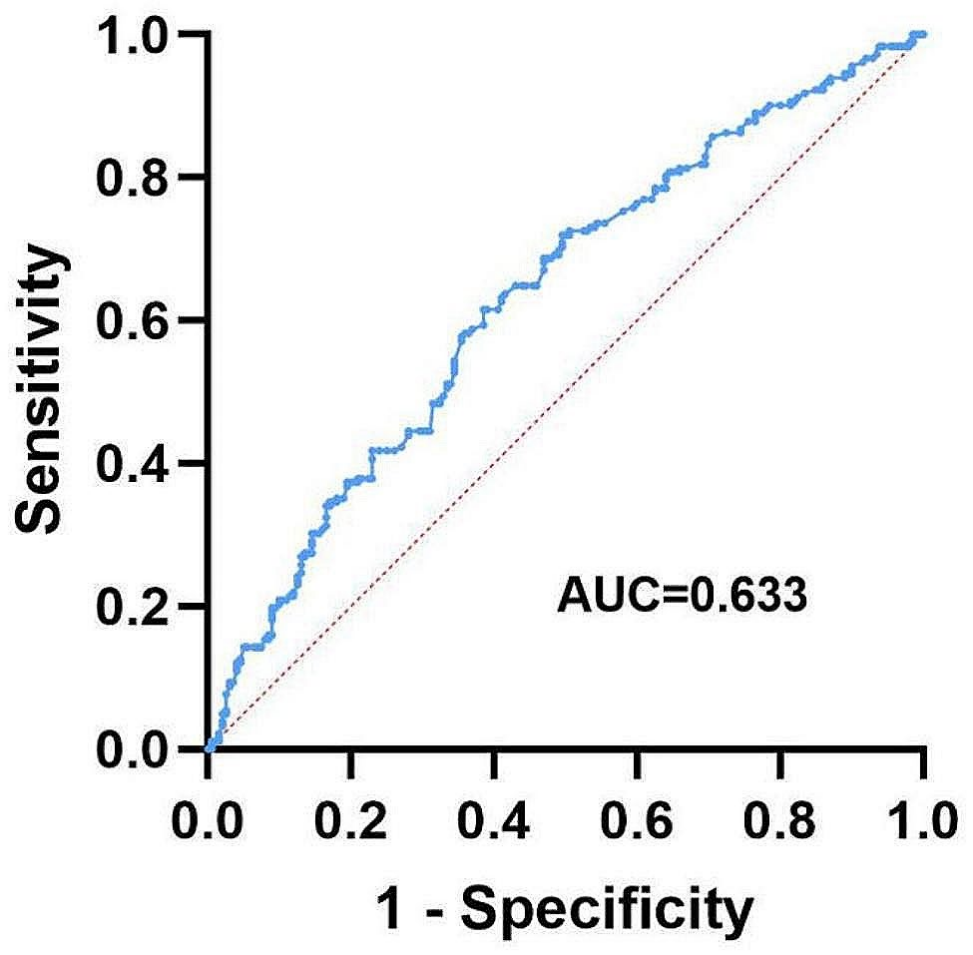
Receiver operating characteristic curve of the interspinous distance associated with diverting ileostomy

Collinearity tests were performed for variables with *p* < 0.05 in the univariate analysis, and collinearity existed between the surgical procedure (LAR and taTME) and anastomosis below the levator ani plane, and the surgical procedure was excluded from the multivariate analysis. The multivariate analysis (Tables 1 and 2) suggested that preoperative radiotherapy (*p* = 0.037, odds ratio [OR] = 2.98, 95% confidence interval [CI] = 1.07–8.30), anastomosis below the levator ani plane (*p* < 0.001, OR = 7.09, 95% CI = 4.13–12.18), and the interspinous distance (*p* = 0.047, OR = 0.97, 95% CI = 0.93-1.00) were independently associated with performing DI. The ROC was used to determine the cut-off value of the interspinous distance to further identify the factors associated with performing preventive stoma. As suggested in Fig. 2, the interspinous distance area under the curve = 0.633, cut-off value = 94.8 mm, and interspinous distance < 94.8 mm were independently associated with performing DI.

Table 3 presents the short-term postoperative status of the patients after surgery. The overall incidence of AL was 6.8%, and the occurrence of AL was not statistically significant (8.2%) compared with that of the stoma group (5.5%) (*p* = 0.288). However, lesser interventions were required in the stoma group on AL occurrence; that is, grade B or C AL occurred less frequently (*p* = 0.04), with a shorter postoperative stay observed in the stoma group (*p* = 0.005).

**Table 3:** Patients’ postoperative conditions

Variable	Total(*n* = 382)	WithoutDI(*n* = 182)	DI (*n* = 200)	pvalue
Anastomotic leak	26(6.8%)	15(8.2%)	11(5.5%)	0.288
Anastomotic leak requiring clinical intervention				
Yes	9(34.6%)	8(53.3%)	1(9.1%)	*0.036
No	17(65.4%)	7(46.7%)	10(90.9%)	
30-day mortality	0	0	0	0
Postoperative hospital stay(d)		7.02 ± 5.22	5.08 ± 2.71	*0.005

*P value obtained using **x**²test,Fisher’s exact text. *P* values are derived from two-tailed tests. *Values are statistical significance (*p*<0.05)

## Discussion

This single-center retrospective study was conducted to explore factors that might influence the choice of DI in laparoscopic-assisted surgery for mid-low rectal cancer and to elucidate the potential benefits of DI in sphincter-preserving surgery. Due to the advances in laparoscopic surgery and the advantages of endoscopic surgery [18, 19], laparoscopic surgery is preferred after preoperative evaluation in patients with rectal cancer at our center. Therefore, only laparoscopic-assisted sphincter-preserving surgery was included in this study. AL is a common complication after sphincter-preserving surgery for low rectal cancer, causing serious morbidity and mortality. A preventive stoma is widely used in mid-low rectal cancer surgery; however, no specific indication for DI exists in any case. Akio Shiomi’s retrospective cohort study reported that DI could be performed in men with rectal cancer and poor nutritional status; however, it is not recommended in women [10]. Mrak et al., in their prospective study, reported that the incidence of AL was lower in the stoma group than in the group without stoma in patients with low rectal cancer (5.8% vs. 16.3%, *p* = 0.0441), with a decreased reoperation rate (1.2% vs. 15%, *p* = 0.021). The multivariate analysis suggested that male sex and no DI were independent risk factors for AL after surgery for rectal cancer [7]. Most studies reported that the choice of prophylactic stoma depends on perception and concern about the factors influencing postoperative AL occurrence. Herein, no significant difference in the overall incidence of symptomatic AL was observed between the stoma and non-stoma groups. However, some cases of AL requiring surgical intervention were observed in the stoma group, with a shorter postoperative hospital stay, which was consistent with several previous studies [6]. This single-center study identified the following factors to be independently associated with performing prophylactic stoma: preoperative radiotherapy, anastomosis below the levator ani plane, and an interspinous distance of < 94.8 mm. These preoperative and intraoperative factors might influence DI selection.

Preoperative radiotherapy is currently widely employed in locally advanced rectal cancer, which curbs tumour progression while also increasing the number of postoperative complications [20]; however, the rational use of DI might lower the corresponding risks. The results of a multicenter randomised trial revealed that in patients treated with chemoradiotherapy or renal replacement therapy before surgery, the stoma could lower the risk of complications caused by preoperative treatment, particularly symptomatic AL [21]. Zhan TC reported that radiotherapy increased the incidence of AL, and DI helped lower the occurrence of radiation-related AL (*p* < 0.001, OR = 6.211) [22]. Lin SC reported that better surgical outcomes could be obtained in patients who undergo rectal cancer surgery receiving preoperative radiotherapy [23]. Herein, preoperative radiotherapy was independently associated with DI selection (*p* = 0.037, OR = 2.98). Some studies have reported that type I collagen plays a crucial role in maintaining the mechanical stability of the anastomotic tissue, and radiotherapy can affect collagen synthesis resulting in poor tissue healing, thereby increasing the risk of AL [24]. DI can lower the severity of rectal surgery-associated complications after radiotherapy and lower the occurrence of abdominal infection after AL and secondary intervention.

TME performed in the narrow, funnel-shaped pelvis makes it challenging to enter the deep pelvis, maintain a clear surgical field, accurately identify the anatomical structures, and perform rectal resection. Reportedly, pelvic stenosis might increase the complications in low rectal cancer surgery [25–27]. Special pelvic factors, such as the sacrococcygeal distance, the pelvic inlet distance, the intertuberous distance, and the interspinous distance, could increase the difficulty of rectal cancer surgery [13, 28]; however, studies on whether the difference in pelvic size in different populations will affect the choice of preventive stoma are scarce [10]. Tsuruta et al. reported that preoperative assessment of the pelvic index in patients with rectal cancer could predict pelvic stenosis, and a pelvic index of ≥ 13 can indicate a low risk of AL preoperatively [29]. Toyoshima et al., in their retrospective study, reported that in 117 patients with rectal cancer undergoing intersphincteric resection, a smaller pelvic inlet plane area significantly increased the risk of AL (*p* = 0.012, OR = 0.998) [30]. At the same time, Yu ZL reported that the pelvic inlet diameter, the interspinous distance, the pelvic outlet diameter, and the intertuberous distance were associated with AL occurrence in the univariate analysis, and pelvic inlet diameter (*p* = 0.018, OR = 0.97) and the intertuberous distance (*P* = 0.008, OR = 0.97) were independent risk factors for AL occurrence in the multivariate analysis [13]. In the current study, patients with a short interspinous distance underwent DI more frequently (*p* = 0.047, OR = 0.97). The interspinous distance was the narrowest in the pelvis, and pelvic stenosis not only results in difficult intraoperative mobilisation, resulting in incomplete TME [31] but also interferes with sealer placement and angle adjustment during distal closure, resulting in poor closure and cutting or multiple closures increasing the risk of AL. The analysis of our center’s findings suggests that DI should be considered while assessing pelvic stenosis by preoperative auxiliary examination.

The levator ani muscle is an important part of the pelvic floor muscle. An ultra-low anastomosis refers to the anastomosis located below the levator ani plane. A prospective multicenter study by Shiomi et al. recommends prophylactic stoma for low anastomosis < 5 cm from the anus and particularly for ultra-low anastomosis < 2 cm from the anal verge [32]. Previous studies have also reported that AL is expected in rectal cancer patients with an anastomotic distance of < 6 cm from the anus, particularly in male patients [7]. AL is closely associated with rectal blood supply and post-anastomotic tension [33]. During rectal cancer surgery, the proximal bowel is pulled to the levator ani plane for anastomosis, and mesangial tension results in poor blood supply to the proximal bowel. In addition to longitudinal traction tension from the bowel, there is transverse traction tension from the pelvic floor muscles for the anastomosis. Splenic flexure mobilisation can only decrease the longitudinal tension and not the transverse tension; therefore, AL is more commonly observed in cases of ultra-low anastomosis. At our center, DI was more frequently performed in patients with anastomoses located below the levator ani plane (*p* < 0.001, OR = 7.09), and appropriate faecal bypass could lower the pressure at the anastomotic site and facilitate tissue healing.

## Limiations and future work

This study has some limitations. First, this was a single-center retrospective study with a small number of patients. A multicenter study might have to be conducted to further verify our results. Second, as the information of some patients was inaccurate, their past history of cancer, personal history of smoking and alcohol consumption, and other nutritional indicators were not included in this study, thereby affecting the experimental results. Despite the limitations of this study, we still provide a cut-off value for pelvic parameters for reference in clinical work. And,using AL as the endpoint to analysis whether pelvimetry plays important role in preventing severe complications is on our research list.

## Conclusion

In conclusion, variations exist in terms of certain clinicopathological features and clinical variables between patients who undergo and do not undergo DI, and these differences affect clinical decision-making. Patients who undergo DI have relatively few occurrences of AL and significantly fewer complications requiring secondary clinical intervention; therefore, DI is selectively recommended. According to our center’s experience, patients who undergo laparoscopic-assisted surgery for mid-low rectal cancer receiving preoperative radiotherapy, with the anastomosis below the levator ani plane and narrow pelvic with an interspinous distance of < 94.8 mm are more likely to undergo DI, and the preoperative doctors should be fully prepared.

## Data Availability

ALL data generated or analyzed during this study are included in this published article.

## Abbreviations

DI: Diverting ileostomy
AL: Anastomotic leakage
LAR: Low anterior resection
taTME: Transanal total mesorectum excision
CT: Computed tomography
